# Experimental demonstration of a trophic cascade in the Galápagos rocky subtidal: Effects of consumer identity and behavior

**DOI:** 10.1371/journal.pone.0175705

**Published:** 2017-04-21

**Authors:** Jon D. Witman, Franz Smith, Mark Novak

**Affiliations:** 1 Department of Ecology and Evolutionary Biology, Brown University, Providence, RI, United States of America; 2 Department of Integrative Biology, Oregon State University, Corvallis, OR, United States of America; Texas A&M University at Galveston, UNITED STATES

## Abstract

In diverse tropical webs, trophic cascades are presumed to be rare, as species interactions may dampen top-down control and reduce their prevalence. To test this hypothesis, we used an open experimental design in the Galápagos rocky subtidal that enabled a diverse guild of fish species, in the presence of each other and top predators (sea lions and sharks), to attack two species of sea urchins grazing on benthic algae. Time-lapse photography of experiments on natural and experimental substrates revealed strong species identity effects: only two predator species–blunthead triggerfish (*Pseudobalistes naufragium)* and finescale triggerfish (*Balistes polylepis*)–drove a diurnal trophic cascade extending to algae, and they preferred large pencil urchins (*Eucidaris galapagensis*) over green urchins (*Lytechinus semituberculatus*). Triggerfish predation effects were strong, causing a 24-fold reduction of pencil urchin densities during the initial 21 hours of a trophic cascade experiment. A trophic cascade was demonstrated for pencil urchins, but not for green urchins, by significantly higher percent cover of urchin-grazed algae in cages that excluded predatory fish than in predator access (fence) treatments. Pencil urchins were more abundant at night when triggerfish were absent, suggesting that this species persists by exploiting a nocturnal predation refuge. Time-series of pencil urchin survivorship further demonstrated per capita interference effects of hogfish and top predators. These interference effects respectively weakened and extended the trophic cascade to a fourth trophic level through behavioral modifications of the triggerfish-urchin interaction. We conclude that interference behaviors capable of modifying interaction strength warrant greater attention as mechanisms for altering top-down control, particularly in speciose food webs.

## Introduction

Concern over increasing levels of human impacts in natural ecosystems has renewed interest in the roles that predators play in food web and ecosystem functioning. As human exploitation is depleting large predators in the ocean [1–3], there is a growing appreciation that many predators have important indirect effects on species lower in the food web by means of their density (Density Mediated Indirect Interactions DMII), behavior (Behaviorally Mediated Indirect Interactions, BMII), and diversity [4–8]. The complexity of tropical and sub-tropical food webs, which includes intraguild predation, compensatory dynamics, and many interacting species, has been presumed to dampen the strength of top predator control in trophic cascades, compared to simple webs with few species [9–11]. Indeed, a review of marine trophic cascades in the tropics found that strong top down control of herbivores is possible, but that effects rarely cascade down to benthic primary producers [12]. Recent studies indicate mixed evidence for trophic cascades in tropical regions as they weren’t detected on coral reefs off Australia [13,14], while McClanahan and Muthiga [15] found that a triggerfish- urchin- algae trophic cascade was widespread on western Indian Ocean reefs.

While there are many reasons why trophic cascades may be context-dependent and the predictions of trophic cascade theory are unsupported in species rich tropical–subtropical communities [9,16] the methodology used to test for trophic cascades may also influence the outcome. For example, with a few exceptions [17], most of the evidence for trophic cascades in tropical–subtropical food webs is based on inverse patterns of abundance between predators and grazers [12–13, 18–19]. There is a surprising lack of TC investigations that use manipulative field experiments to directly test whether the processes inferred from patterns in tropical–subtropical subtidal habitats are actually causal. This is true for our study system, the Galápagos subtidal, as well, where Sonnenholzner [20] and Edgar et al. [21] used surveys of benthic invertebrate and fish populations to infer that consumptive trophic cascades occur in a tri-trophic pathway where either hogfish, other carnivorous fishes and/or lobsters controlled the abundance of sea urchins, in turn releasing benthic algae from grazing.

As a consequence of the prevalence of survey-based analyses, which do not experimentally establish casual links in the field, the ability to make strong inferences about trophic cascades and the mechanisms controlling primary producers in tropical–subtropical regions is limited. Similarly limiting is the fact that efforts to build synthetic trophic cascade theory for diverse food webs have relied on few studies that have simultaneously evaluated DMII’s and BMII’s [22–24] which in the ocean have largely focused on lower trophic levels. Although higher trophic level consumers such as predatory fishes and sharks are most threatened by human activities [3, 25], few experiments have been performed where these predators have the potential to influence trophic cascade strength [26, 27]. The influence of behaviorally-mediated indirect interactions have not been investigated in Galápagos marine food webs.

We addressed these information gaps by testing for the effects of consumer species identity and behavior on the strength of top down control in an interaction web of sharks, sea lions, triggerfish, hogfish, sea urchins and benthic algae in the Galápagos Islands, a region of high consumer diversity with 16 species of urchin predators (S1 Table) [28, 29]. The Galápagos Marine Reserve (GMR), in which we performed our study, presents a rare opportunity to test theory about the influences of consumer identity and behavioral interactions within a diverse, sub-tropical food web because higher trophic level predators such as sharks, sea lions and large fish are still abundant. The main questions we posed were: 1. Do Trophic Cascades (TC’s) occur in this diverse sub-tropical system? 2. If so, do they depend on consumer (predator and herbivore) species identity? 3. Do behavioral interactions dampen top down control of primary producers?

**Fig 1:**
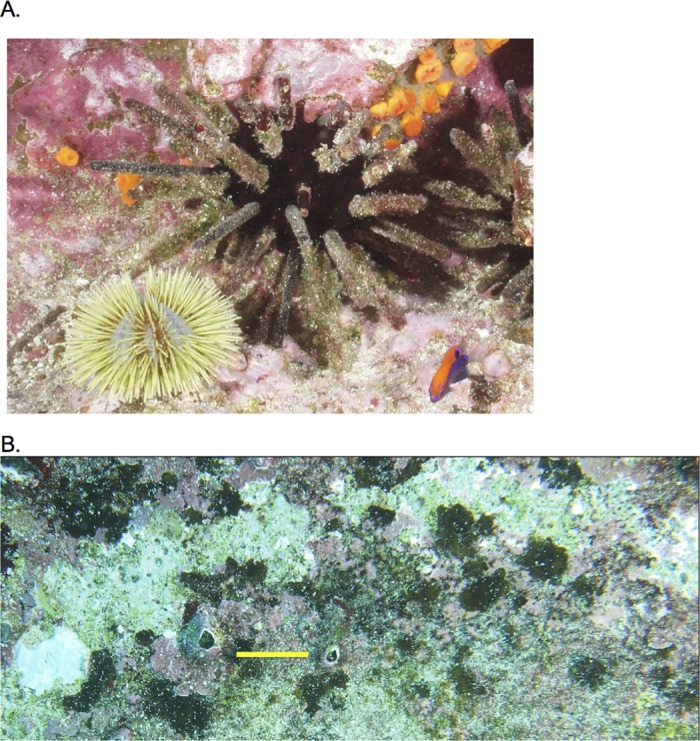
(**A**) **Green (*Lytechinus semituberculatus*) and pencil (*Eucidaris galapagensis*) sea urchins on the exposed rocky substrate, 8 m depth, Galápagos Islands and (B**) **close-up of a patch of substrate on the bases grazed by pencil urchins in the TC experiment.** Pink areas are crustose coralline algae which is consumed by both species. Orange organisms are an ahermatypic coral *Tubastrea* sp. Scale bar in (**B**) is 2.0 cm. The grazed area is a light-colored crescent shaped patch approximately 2.0–3.0 cm wide extending across the upper half of the photograph. These areas were measured as a percent of the substrate grazed by sea urchins in the TC experiments. Also shown are crustose coralline algae (pink), fleshy encrusting algae *Hildenbrandia* sp. (brown), green algal turf, and diatoms (light brown).

Two problems confront an experimentalist trying to incorporate the complexity of diverse food webs into tests of trophic cascades in the field. One is that it is difficult to assess the effects of multiple consumer species interactions in species-rich polycultures due to the infeasibility of running an experiment with all *n*! factorial combinations of many consumer species [30, 31]. Secondly, many consumers are large and highly mobile, precluding the typical experimental approach of enclosing or individually excluding them using cages [25, 32, 33]. With these problems in mind, we adopted a hybrid approach to test for trophic cascades using experimental manipulations where grazers (sea urchins) were restricted by fences yet were vulnerable to predation from a speciose guild of predatory fish in the water column. We refer to this as an open experimental design [32, 33] since unconfined predatory fish could move in and out of the treatments, attacking the sea urchins, affecting their consumption of benthic algae, and interacting with each other and with top predators. This enabled us to evaluate both DMII’s and BMII’s, and to make strong inferences about the presence of a trophic cascade by quantifying each of its key components [34]. We focused on the two most abundant sea urchin species in the Galápagos subtidal, the pencil urchin, *Eucidaris galapagensis*, and the green urchin, *Lytechinus semituberculatus* (Fig 1A) that are known to be strong algal grazers [35–37]. A series of experiments were also performed with tethered sea urchins to examine spatial variation in top-down control and to test hypotheses about prey species and size selection by predatory fishes.

## Materials and methods

This field research was conducted under a permit from the Galápagos National Park Directorate.

### Top down control of pencil urchins–tethering experiments

Top-down control of sea urchin populations was investigated at multiple sites by urchin tethering experiments. The tethering procedure involved tying live sea urchins by their test without puncturing them to a small rock or weight, enabling them to move around on their string tethers [38]. We conducted 8 trials using small pencil urchins (2.3–2.6 cm Test Diameter, TD) initially, placing them on the natural rock substrate at 10–12 m depth at 4 sites: Isla Champion, Rocas Gordon, Isla Baltra South and Guy Fawkes (S2 Table) [39]. We used large pencil urchins (4.0–5.0 cm TD) in 2 additional tethering trials at the Baltra South site (150 m south of the TC experimental site, 12 m depth). To discern the influence of predator species identity, predation on sea urchins was monitored by time-lapse cameras. In 2007–2008 the experiments were filmed by a Canon D20 digital camera in an Aquatica metal housing, equipped with an external remote and Canon timer. Photographs were taken at 1 minute intervals during daylight trials and at 2 minute intervals during overnight experiments, using a strobe for illumination. In 2009–2012 the experiments were monitored by GoPro cameras photographing at 1 or 2 second intervals, occasionally using them in video mode. Attacks on the tethered urchins by fish were recorded in the time-lapse images which enabled the predator to be identified to species. This method also revealed urchins that escaped from their tethers, eliminating error caused by including urchin disappearances in the mortality estimates. The sequence of tethering experiments progressed from the initial experiments that examined spatial variation in predation on small pencil urchin prey to subsequent experiments with large urchins. These were followed by explicit tests of prey size and prey species selection.

### Diel variation in pencil urchins

Diel variation in urchin density on the rocky substrate was evaluated by counting urchins visible in the time-lapse images of a 1.3 m^2^ area peripheral to the urchin tethering experiments conducted at the Baltra South Site from June 27–28, and July 3-4^th^, 2008. Photographs were taken at 2 minute intervals with the Canon time-lapse camera system (as above) for 24 hours. Counts of pencil urchins were made at one hour intervals; green urchins were not present during the camera deployments. All non-cryptic urchins in the area could be detected in the high-resolution (12 megabyte) images.

### Size selective predation on urchins

Two experiments were conducted to test for size selective predation on green and pencil urchins. The experiment with green urchins began on January 8, 2013 when 26 small (2.23 cm TD, 0.42 SD) and 26 large (4.10 cm TD, 0.50 SD) urchins were tethered and placed on top of the algal covered bases used for the TC experiment (below) so that there were 2 urchins of each size category on 13 bases. Predation was monitored at 2 second intervals during daylight hours with one GoPro camera trained on the entire experiment and two cameras mounted on short tripods (0.5 m high) on the sides of the experimental area (~ 10 x 5 m spatial extent).

The test with pencil urchins began on January 9, 2013 using the same experimental design except that 32 small (2.25 cm TD, 0.38 SD) and 31 large (4.44 cm TD, 0.68 SD) urchins were tethered and placed on 14 bases with 2 large and 2 small individuals on every base except for one, which had one large and two small urchins on it. Six small and 5 large urchins escaped from their tethers during the first day of the experiment, reducing replication to 26 urchins per size group.

### Trophic cascade experiments

The hypothesis that predation on urchins has a positive indirect effect on benthic algae by releasing algae from urchin grazing was tested by the open experimental design with treatments where urchins were exposed to fish predation and predatory fish were unconfined. In these experiments, circular concrete bases (0.31 m ^2^ area, 10 cm thick) with embedded bolts were used to facilitate fence and cage attachment underwater. Bases were placed at 10–12 m depth over an area of approximately 30 m by 10 m on a sloping ledge at the NE corner of Baltra Island (S2 Table) in June 2011 and were left for 12 months to enable natural algal assemblages to colonize. Fences and cages were made of plastic coated steel mesh (Aquamesh, 2.5 cm mesh, 53.0 cm diameter, 12.0 cm high) enclosing a footprint of algal-covered substrate measuring 0.22 m^2^. The algal community on the bases at the start of the experiment was qualitatively similar to that on the natural substrate at the Baltra site and at Islote Caamaño, Galápagos [37]. It was largely composed of a pavement of crustose coralline algae with patches of red and green algal turf, diatoms, erect coralline algae, fleshy crustose algae and *Ulva sp*. The main variable quantified as a measure of a trophic cascade strength was the percent of the algal substrate consumed by urchins in the different treatments (Fig 1B). Changes in the composition of the algal community were not assessed.

The experiment consisted of two treatments and a control: a predator exclusion cage containing 4 urchins, a predator access treatment (fence) containing 4 urchins, and a control cage lacking urchins, with n = 6 replicates per treatment. Treatments were randomly assigned to the algal covered bases. The densities used in the experiments were representative of natural urchin densities, which for pencil urchins can attain maximum densities of 12.9 individuals per 0.25 m ^2^ at Isla Champion and 26.5 per 0.25 m ^2^ for green urchins at Las Tjeretas, San Cristobal Island (J. Witman *unpublished data*) [36–38]. Green urchins co-occur with pencil urchins (Fig 1A), although they are generally more abundant at depths shallower than at the 10–12 m depths of the TC experiments [36, 40]. The urchins were placed in holding cages without food for 24 hours prior to the start of the experiments. Two GoPro II cameras (GoPro Inc., San Mateo, CA, USA) mounted on surveyor’s tripods were used to film the bases from a height of 2 m above the substrate. Pencil urchins averaging 3.9 cm TD (0.3, Standard Deviation, SD, n = 24) were used in the first trial beginning on 11:46 on June 23, 2012 which ran for 8 days. Predation on the urchins and behavioral interactions among all consumers were quantified by time lapse photography at 1 second intervals during daylight (approximately 06:30–17:45 hours) throughout the entire period. Divers replaced the camera batteries at 4 hr. intervals.

A second TC experiment was performed using the same design but with green urchins, using individuals averaging 3.8 cm TD (0.2 SD, n = 24). This TC experiment began on July 13, 2012 at 12:20 and ran for 7 days. Four green urchins were used per treatment and they were monitored by the same time-lapse imaging methods. The amount of algae grazed from the substrate by urchins was measured from digital photographs (12-megapixel resolution) of the bases taken with a camera framer prior to and at the end of the experiments using NIH ImageJ software.

Temperatures were recorded at 5 minute intervals throughout each TC experiment by an Onset Tidbit data logger (Onset Computer Corporation, Pocasset, Massachusetts, USA, ± 0.01° C precision) attached to one of the control bases.

The high frequency photographic record of the TC experiments enabled apparent handling times to be directly measured in seconds as the time from when predatory fish (blunthead, *Pseudobalistes naufragium* or finescale *Balistes polylepis* triggerfish) first contacted the urchin until it was abandoned. The behavioral effects of top–predators and hogfish interference on triggerfish feeding were also directly quantified at 1 second intervals from the time-lapse images. This was done while urchins remained as prey in the predator access treatments (fences), which was the first 21 hours of the pencil urchin experiment and for 6 of the 7 days of the TC experiment with green urchins. A total of 58,920 time-lapse images (29,460 per camera) were analyzed during the pencil urchin experiment and 247,436 images during the green urchin experiment. Interference was considered to encompass behaviors that caused fish to lose time when avoiding or fighting with competitors, or when a prey was stolen (i.e. kleptoparasitism) [41]. For these direct measurements we considered hogfish interference behavior as the close following or circling of a triggerfish, with close defined as within 2 triggerfish body lengths (S1 Fig, S1 Video). These behaviors were also recorded during the TC experiment with green urchins.

To quantify the cascading effects by which hogfish, sharks, and sea lions were observed to alter triggerfish foraging behavior and thereby indirectly affect grazing rates by altering pencil urchin abundances, we fit a model to the time course of pencil urchin survivorship in the predator access treatment [42]. In this model the number of urchins (*U*) eaten per triggerfish (*P*) per time-step (Δ*t* = 1 min.) was described by *U*_Δ*t*_ *= U*_*t*_ *(*1*-exp*[*F*(*U*_*t*_, *H*_*t*_, *T*_*t*_) *Pt* Δ*t*]), where the triggerfish foraging rate was a function *F* of urchin, hogfish (*H*) and top-predator (*T*, sea lion and shark) abundances at time *t*. The time-lapse photo observations were used to quantify the effects of hogfish and top-predators on triggerfish foraging rates using a Beddington-DeAngelis-like functional response of the form *F*(*U*_*t*_, *H*_*t*_, *T*_*t*_) = *a /* (1 + *ahU*_*t*_ + *bH*_*t*_ + *cT*_*t*_) [43–45]. Here the parameters *a* and *h* respectively represent the per capita attack rate and the effective handling time (including both pre- and post-ingestion rate-limiting processes) with which triggerfish consume urchins, and the parameters *b* and *c* respectively represent the behavioral interference rates with which hogfish and top-predators (sharks and sea lions) affected the strength of the triggerfish-urchin interaction. The model was fit to the pencil urchin trophic cascade experiment (total urchins summed across all bases) by maximum likelihood using a binomial likelihood to describe the probability of observing an urchin attack within each time interval [44]. We did not fit the model to the green urchin cascade experiment since no urchins were eaten.

We assumed a model of an equivalent form in estimating the per capita grazing rates of each of the two urchin species (*g*), letting the area of algae remaining at the end of an experiment (*A*_*t* = *T*_) be a function of the algal area at the start of the experiment (*A*_0_ = 0.22 m^2^) and the number of urchins to which algae were exposed over time, *A*_*T*_ *= A*_0_ *exp*[*-gUT*]. That is, we assumed that urchin grazing was proportional to algal cover and that algal growth after grazing was trivial over the course of the experiment [45]. Grazing rates were estimated assuming a lognormal likelihood using each of the *n* = 6 base-specific grazing rates per treatment [44]. Confidence intervals were estimated by the *BC*_*a*_ bootstrap procedure [46].

### Species selective predation on urchins

As the trophic cascade experiments were indicating differential rates of predation on pencil and green urchins, additional tethering experiments were conducted in which both urchin species were simultaneously exposed to predation to test the hypothesis that predation did not differ between urchin species. In the first experiment, 23 urchins of each species were individually tethered to small rocks and placed on natural ledge and cobble substrates in each of two areas 4–5 m apart on July 3, 2012. This prey choice experiment was conducted at the same site as the TC experiments. The experiment was replicated at the Baltra South site on July 19, 2012. In this second experiment, pencil and green urchins were tethered and placed on the natural substrate in two areas 5 m apart with 25 of each species in one area and 27 of each in the second. In both experiments, predation on the urchins was video recorded for the first 2.5–3.0 hours by GoPro cameras and subsequently monitored during the day by divers at 4 hour intervals.

## Results

### Top down control of pencil urchins–tethering experiments

**Fig 2:**
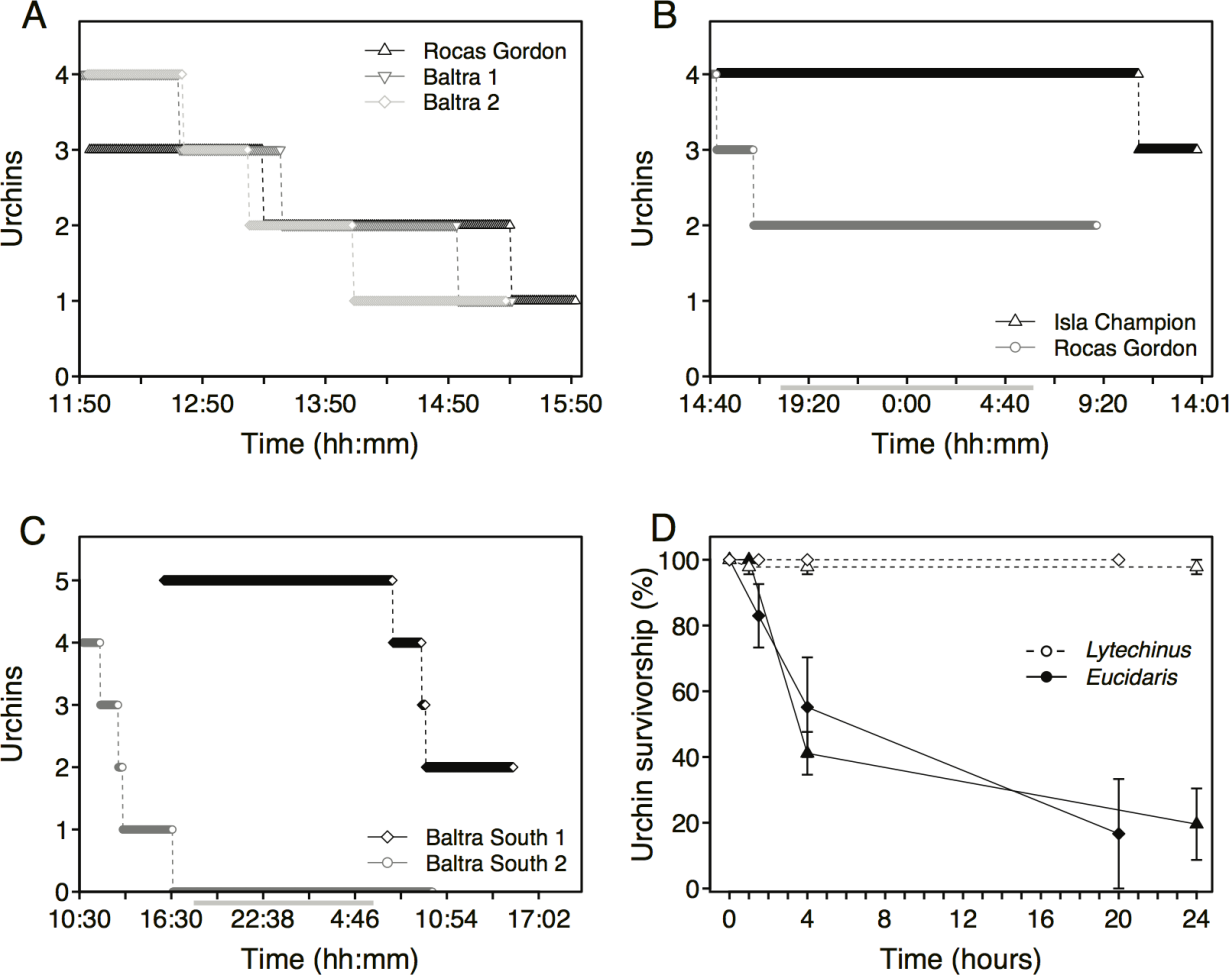
(A-B) Pencil urchin (*Eucidaris*) survivorship and prey selection from tethering experiments. Data points represent the number of surviving small urchins at 1 minute intervals in A-B, and the number of large urchins surviving at 2 min. intervals in C., all from time-lapse photographs. Gray bars below the x-axis in B and C represent night time hours of darkness. Experiments in A were conducted at Rocas Gordon on May 18, 2007 and at Baltra South on May 22 and May 23, 2007, and in B at Isla Champion (January 11, 2008, open triangles) and Rocas Gordon (January 7, 2008 open circles). (C) Survivorship from trials where blunthead and finescale triggerfish consumed the large urchins. The diamond symbols represent a trial initiated on June 27, 2008 while the circles represent a trial begun on July 3, 2008. (D) Average urchin survivorship from two trials of prey selection experiments performed with tethered green urchins (open diamond and triangles) and pencil urchins (black diamond and triangles) placed next to each other on the substrate.

Time-lapse images indicated that small pencil urchins were eaten by adult hogfish *B*. *diplotaenia* at 3 of the 4 sites where tethering experiments were conducted (Fig 2, S2 Fig). Urchin survivorship ranged from 25% at Rocas Gordon to 100% in two trials at Guy Fawkes (Fig 2, survivorship at Guy Fawkes not plotted). Average survivorship of small urchins incurring hogfish predation was 66.6% at 207 minutes elapsed time and 63.5% at the end of the experiments (n = 4 sites). There was significant site variation in the survivorship of small urchins at both 207 min. and at the end of the trials (*F* = 165.96, *P* < 0.0001, *F* = 11.03, *P* < 0.021, respectively, 1-way ANOVA, both with 1,3 df, data arcsine transformed). *B*. *diplotaenia* was responsible for all predation in these experiments, and no predation occurred at night (Fig 2B).

In contrast, large pencil urchins (4.0–5.0 cm TD) were readily eaten by blunthead and finescale triggerfish. These two fish species were responsible for all predation in the tethering experiments at the Baltra South site (Fig 2C, S2C and S2D Fig). Average survivorship of large urchins exposed to predation during the 24 hr. trials was low at 20.0% (28.2 SD), with no urchins surviving triggerfish predation in one trial. As in the small urchin tethering experiments, no predation occurred at night (Fig 2C). Hogfish were observed to interfere with triggerfish foraging during 40–50% of these predation events (S3 Table, S3C Fig).

### Diel variation in pencil urchins

**Fig 3:**
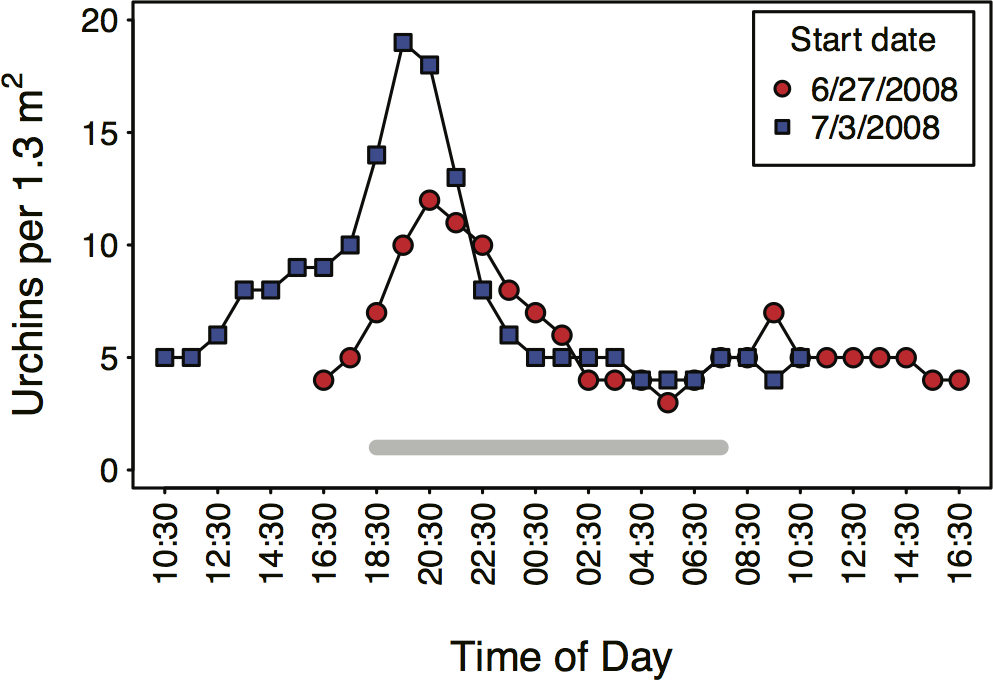
Diel patterns of pencil urchin density at the Baltra South site. Data represent the number of urchins per 1.3 m ^2^ on the exposed substrate as determined by counting urchins in time-lapse photographs in 2 trials beginning on June 27 and on July 3, 2008. The grey bar below the x axis represents night hours. Note the rapid increase in urchin abundance during the early hours of night and that urchins are exposed on the substrate during the day.

Replicated time-lapse camera deployments revealed pronounced diel variation in the abundance of pencil urchins (Fig 3) at the Baltra South site. Urchins were exposed on the surface of the rock substrate during the day in densities of 3.0–10.0 per 1.3 m^2^, increasing during the early hours of night beginning at 19:30 on June 27^th^ and at 16:30 on July 3^th^. Maximum densities of 12–19 urchins occurred from 19:00 to 21:00, then declined over the next 3–6 hours to near constant densities of 4–5 individuals per 1.3 m^2^.

### Size selective predation

Tethering experiments rejected the hypothesis of no size selective predation on pencil urchins, as the percent of large urchins consumed by triggerfish was 52 times higher than that of small urchins (38.4 large versus 0.73% small urchins, Fisher’s exact test, *P* < 0.01, 1, 52 df). With one exception, triggerfish were responsible for all of the predation on urchins in these experiments as indicated by time-lapse images of the experiment and by pencil urchin tests cleaved in half, a trademark of triggerfish predation (S2C Fig). The exception was one small pencil urchin consumed by a hogfish. There was no predation on tethered green urchins of either size group. There was, however, one unsuccessful attack when a hogfish bit a large green urchin but broke off the attack immediately after contacting it.

### Prey choice

Replicate experiments with pencil and green urchins tethered side by side on the natural rock substrate indicated that triggerfish preferred pencil over green urchins (Fig 2D, S2E Fig). For example, the average survivorship of pencil urchins was 78.2% lower than that of green urchins during the first trial. The only predation on green urchins occurred 15 minutes into the 24 hr. experiment when a hogfish attacked and swam off with 1 tethered green urchin. Only one of 37 pencil urchins consumed in this trial was eaten by a hogfish, 420 minutes into the experiment. The other 36 were eaten by blunthead triggerfish as observed, or by either triggerfish species as indicated by cleaved but still tethered urchin remains (S2C Fig). The second trial at Baltra South yielded similar results, with no green urchins consumed but 34 of 42 pencil urchins tethered next to them eaten by blunthead triggerfish (videotaped), or by either triggerfish species as indicated by cleaved urchin remains. Repeated measures ANOVA indicated a significant difference between the survivorship of the two urchin species in the first (*P* < 0.0005, *F* = 29.64) and second trials (*P* = < 0.003, *F* = 4.68, both with 3,6 df) on arcsine transformed data. There were also significant time and time-by-species effects in both trials (*P* < 0.01).

### Trophic cascade experiment: Pencil urchins

**Fig 4:**
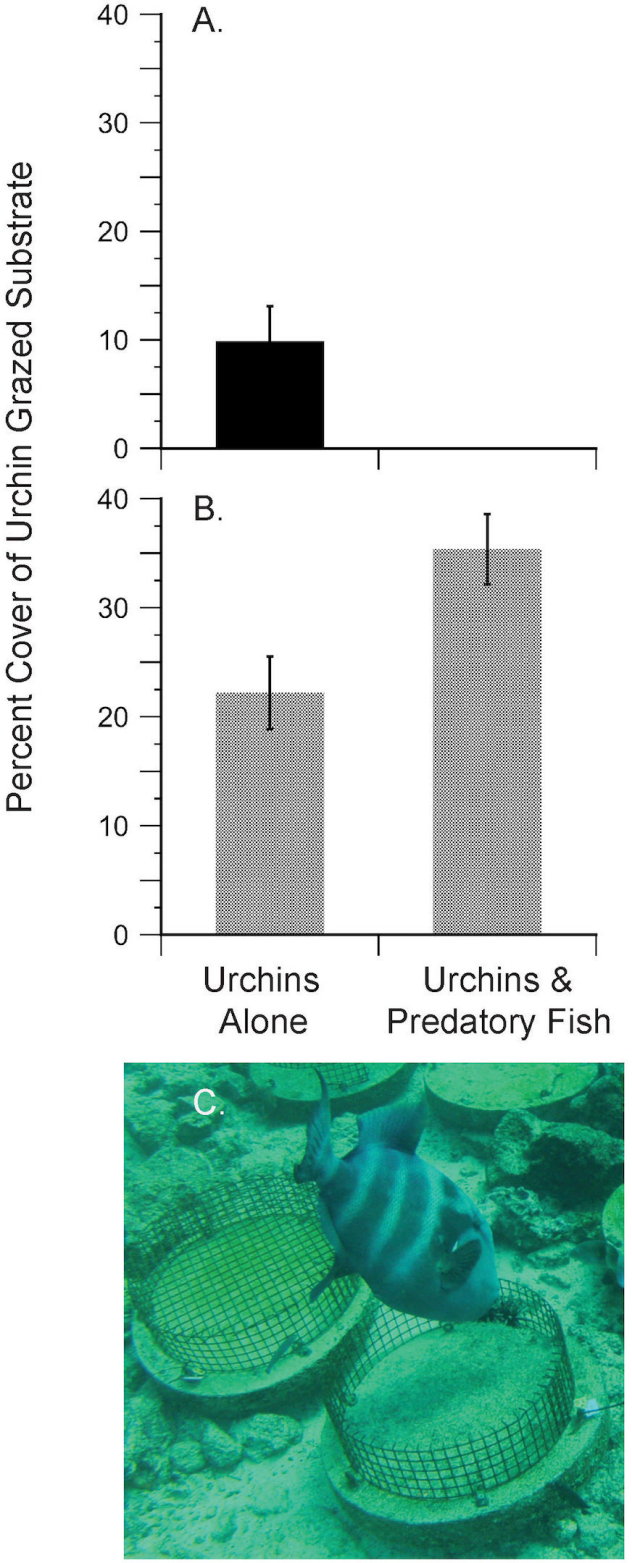
**Results of the trophic cascade experiments with pencil urchin** (**A**) **and green sea urchin prey** (**B**). Bars represent the average percent (+/- SE) of algal covered substrate grazed by urchins in 8 days (A) and 7 days (B). Note that no algae was grazed by pencil urchins in the treatments accessible to predatory fish because they were eaten by triggerfish, providing evidence of a tri-trophic cascade. Green urchins were unregulated by predation and consumed large amounts of algae (B). No algae was lost in the controls (not shown) indicating that environmental factors did not influence the treatment results. (C) Photo from the time-lapse record showing a blunthead triggerfish attacking a pencil urchin during the TC experiment in a fence (predator access) treatment on the morning of June 24, 2012. A caged control is shown to the left of the fence treatment.

A large blunthead triggerfish began feeding on pencil urchins in the fence treatments shortly after the start of the experiment (Fig 4C, S2F Fig). This predation continued for two hours and thirty-four minutes whereby 21/24 of the total urchins in the fence treatments were consumed. Three urchins remained exposed in the fence treatments overnight (18:00 June 23, 2012 to 06:30 next morning) revealing a lack of nocturnal predation. These remaining urchins were eaten by a different (smaller) blunthead triggerfish during the morning of June 24^th^ between 08:01 and 08:50. Predation of pencil urchins by triggerfish was estimated to occur at a per capita attack rate (*a*) of 0.240 (0.405 Standard Error, SE) urchins per triggerfish per minute per urchin available and an effective triggerfish handling time (*h*) of 2.53 (1.084 SE) minutes per urchin in the absence of interfering hogfish and top predators.

There was no mortality of pencil urchins in the predator exclusion cages where they were free to graze on benthic algae. Pencil urchins grazed an average of 9.9% (8.0 SD) cover of benthic algae off the substrate in 8 days (Fig 4A), with grazing scars being readily recognized as irregularly shaped, wide patches of bare substrate (Fig 1B). Since all the pencil urchins in the fence treatments were eaten by predatory triggerfish within 21 hours, no algae were grazed off the substrate by urchins in these predator access treatments. Algal cover remained unchanged in the control cages throughout the experiment indicating that no other factors contributed to the loss of algae during the TC experiment (data not shown). One-way ANOVA on the arcsine transformed percent cover of urchin grazed algae indicated a significant difference between the predator exclusion (cage) versus predator access (fence) treatments (*F* = 66.61, 1,10 df, *P* < 0.0001), providing experimental evidence of a 3-level consumptive trophic cascade from triggerfish to pencil urchins to benthic algae. In the absence of predation, the grazing by pencil urchins was estimated to occur at a per capita rate (*g*) of 2.30 x 10^−6^ percent algae per urchin per minute per algae available (Table 1).

**Table 1:** Effects of predators on the per capita grazing rate of pencil (*Eucidaris galapagensis*) and green (*Lytechinus semituberculatus*) urchins (*g—*algae per algae per urchin per minute) in the presence (fenced treatment) or absence (cage treatment) of top-predators.

Urchin Species	Predators	Grazing Rate (g)	Lower 95% CI	Upper 95% CI
Pencil	present	*	*	*
Pencil	absent	2.30 x 10^−6^	1.30 x 10^−6^	5.10 x 10^−6^
Green	present	1.11 x 10^−5^	7.40 x 10^−6^	1.22 x 10^−5^
Green	absent	6.40 x 10^−6^	4.50 x 10^−6^	8.40 x 10^−6^

The symbol, *, indicates that all urchins were consumed within 21 hours, hence no algae was grazed by the end of the experiment.

### Trophic cascade experiment: Green urchins

In striking contrast to the experiment with pencil urchins, no green urchins were consumed during the TC experiment when they were used as prey. Predator occurrence data from the time-lapse cameras indicated this was not due to a lack of urchin predators during the experiment (Table 2). In the absence of predation, green urchins were able to graze large amounts of benthic algae off the substrate, grazing an average of 35.3% (7.95 SD) of algal cover in the fenced treatments and 22.2% (8.22 SD) in the caged treatments in 7 days (Fig 4B). This grazing corresponded to per capita rate (*g*) estimates of 1.11 x 10^−5^ and 6.40 x 10^−6^ percent algae per urchin per minute per algae available in the presence (fence treatment) and absence (cage treatment) of predators respectively (Table 1).

**Table 2:** The percent of time that consumer species were present in the experimental area while sea urchin prey remained in the trophic cascade experiments (21 hours in the pencil urchin, TC experiments, 168 hours in the green urchin experiments, respectively). The percent of time that a consumer occurred in the area was calculated as a percent of the total time that experiment was recorded by time-lapse photography, which was 29,460 seconds in the pencil urchin TC and 247,436 seconds in the green urchin TC experiment. Predator residence times were analyzed for 6 out of 7 total days of the green urchin experiment. “Other triggerfish” represent combined occurrences of *Sufflamen verres* and *Balistes niger*. Shark species were pooled in the green urchin TC experiment. Species codes are: (H) = hogfish, (T) = triggerfish, (S) = shark, (R) = ray, (SL) = sea lion.

Species and Species Code	% Total Time During Pencil Urchin TC Experiment	% Total Time During Green Urchin TC Experiment
*Bodianus diplotaenia (H)*	32.100	21.700
*Balistes polylepis (T)*	13.360	0.400
*Pseudobalistes naufragium(T)*	0.013	1.740
Other triggerfish	0.010	6.600
All sharks	0.482	0.160
*Trianedon obesus (S)*	0.044	0
*Carcharhinus galapagensis (S)*	0.092	0
*Sphyrna lewinii (S)*	0.340	0
*Aeobatus narinari (R)*	0	0.270
*Zalophus wollebaeki* (*SL*)	1.140	0.090

### Trophic cascade experiment: Hogfish and top-predator effects

**Fig 5:**
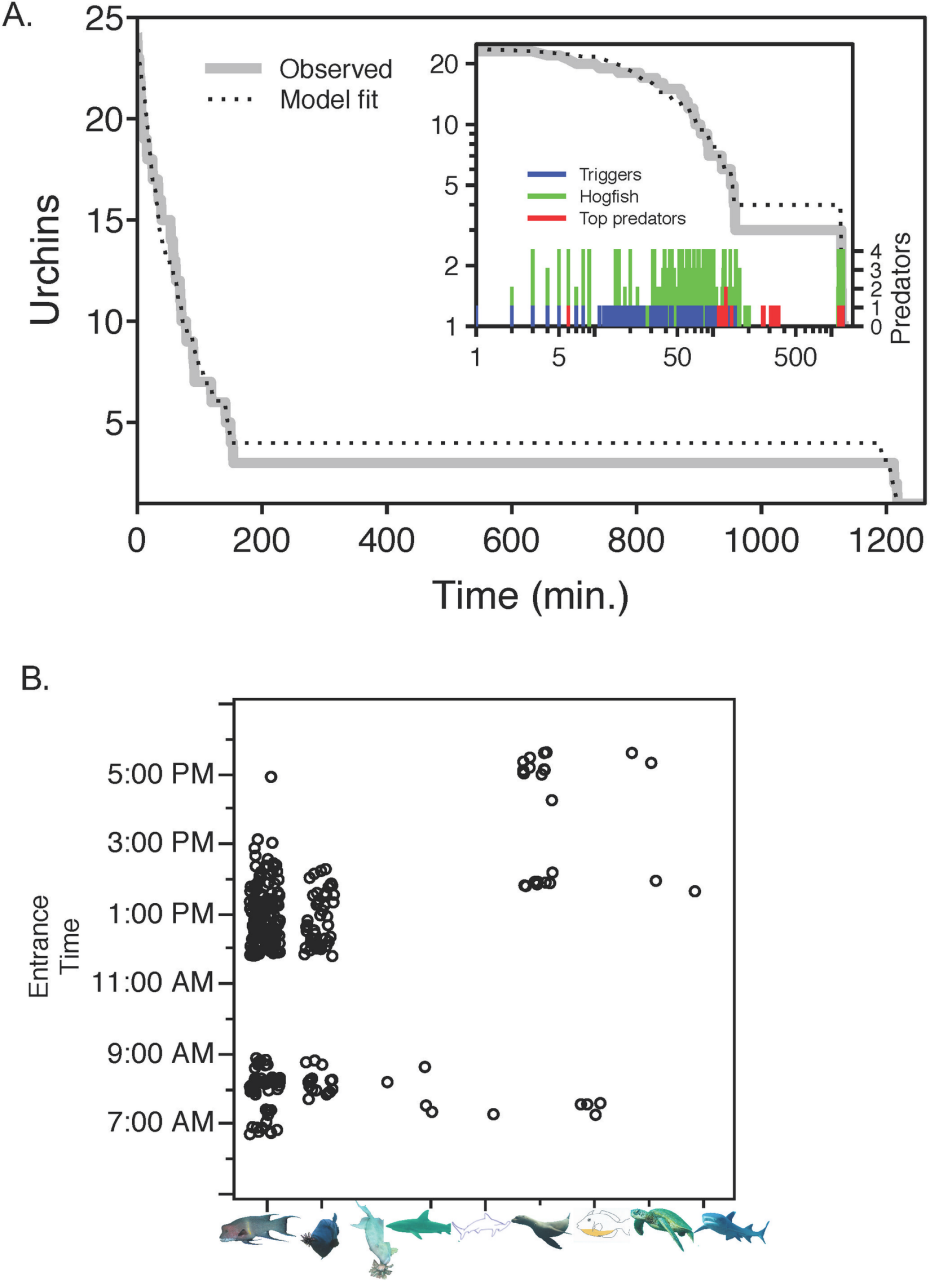
(**A**) **Observed and model-fit pencil urchin survivorship during the initial 20.5 hours of the TC experiment and** (**B**) **consumer co-occurrences during initial 21 hours of the 8 day pencil urchin TC experiment (11:46 until ~ 09:00 the next morning) when blunthead and finescale triggerfish were present.** All of the exposed urchins in the predator access treatments were eaten by blunthead triggerfish (Fig 4C). Inset graph shows the same survivorship results plotted on a logarithmic scale to show the occurrence of triggerfish, hogfish and top-predators (sharks and sea lions). (**B**) Data points represent the first time point that a species was observed in the time-lapse record (entrance time in seconds, y axis) as an indicator of consumer species overlap. Actual consumer residence times are presented in Table 2. Predator images below the x-axis represent hogfish, blunthead triggerfish, finescale triggerfish, Galápagos sharks, hammerhead shark, sea lions, yellow bellied triggerfish, green turtles, and white tip sharks (from left to right).

Eight species of predators were present during the initial 21 hours of the pencil urchin TC experiment (Table 2). Spanish hogfish *Bodianus diplotaenia* were present for 32.1% of the time, followed by blunthead triggerfish at 13.3% of the experimental duration. As roving predators, sharks (including Galápagos, scalloped hammerhead, and white tip sharks) occurred for 0.48% and sea lions for only 1.1% of the time over the approximately 300 m ^2^ study area. Six of the 8 consumers co-occurred with the species responsible for urchin predation during the experiment which were the triggerfish *Balistes polylepis* and *Pseudobalistes naufragium* (Fig 5A and 5B**)** A similar assemblage of predators occurred during the green urchin TC experiment, with hogfish dominating at 21.7% of the time (Table 2). Finescale triggers were more frequent than blunthead triggers, while sharks and sea lions occurred sporadically as in the previous experiment (Table 2).

Hogfish were observed to interfere with blunthead triggerfish in 87.5% of triggerfish attacks on pencil urchins. This increased the average apparent handling time of the triggerfish 2.5-fold, from an average of 40.0. (23.5 SE) seconds without to 101.6 (8.1 SE) seconds with hogfish interference (1-way ANOVA, F = 15.04, P < 0.001, 1, 19 df, data log transformed, n = 3 and 18 respectively). The behavior consisted of hogfish closely circling the feeding triggerfish (S1 Fig, S1 Video), often attempting to bite the urchin prey and interrupting the triggerfish while feeding on the urchin. The impact of hogfish on triggerfish feeding rates corresponded to a model-estimated per capita interference rate (*b*) of 1.086 (2.404 SE) urchins per urchin per hogfish. With all 24 urchins unconsumed, the presence of a single hogfish thereby contributed to a reduction in triggerfish feeding rates of 0.033 urchins per triggerfish per minute (Table 3). In 61.1% (11/18) of the instances of hogfish interference, the hogfish fed on the pencil urchin remains after the triggerfish abandoned it.

**Table 3:** The estimated number of urchins consumed per triggerfish (baseline) when 24 urchins were present and the amount by which the presence of a single hogfish or top-predator (shark or sea lion) reduced this feeding rate from the baseline level (no other predators present).

Interference Effect	Feeding rate (*U*_Δ*t*_)	Difference
Baseline	0.724	NA
Hogfish	0.757	0.033
Sharks or sea lions	1.134	0.409

During the trophic cascade experiment with green urchins, hogfish followed a finescale triggerfish closely on 8 occasions and a blunthead triggerfish once when they were near to the treatments. On average, hogfish circled triggerfish closely for 56.6 seconds (74.0 SD).

Sea lions were actively foraging for fish throughout the water column immediately above the trophic cascade experiments for more than 5 minutes and 3 minutes during the pencil and green urchin experiments respectively (Table 2). This caused fish in the water column to scatter and dive toward the bottom, momentarily suspending fish predation and fish herbivory. In one instance, a blunthead trigger broke off its predation on a pencil urchin during the experiment, dropping the urchin as the sea lion and prey-fish were diving toward the bottom, suggesting that it was a startle response to an unexpected risk cue (S3 Fig). In addition, a sea lion displayed ambush predation, lying on the bottom adjacent to the experimental treatments, looking up at the water column then rapidly swimming up to chase fish. This behavior occurred three times between 17:04 and 17:35 on June 23, 2012. Overall, the resulting impact of top-predators on triggerfish feeding rates corresponded to a model-estimated per capita interference rate (*c*) of 9.11 (24.28 SE) urchins per urchin per top-predator (S4 Table). With all 24 urchins unconsumed, the presence of a single top-predator thereby contributed to a reduction in triggerfish feeding rates of 0.409 pencil urchins per triggerfish per minute (Table 3).

## Discussion

**Fig 6:**
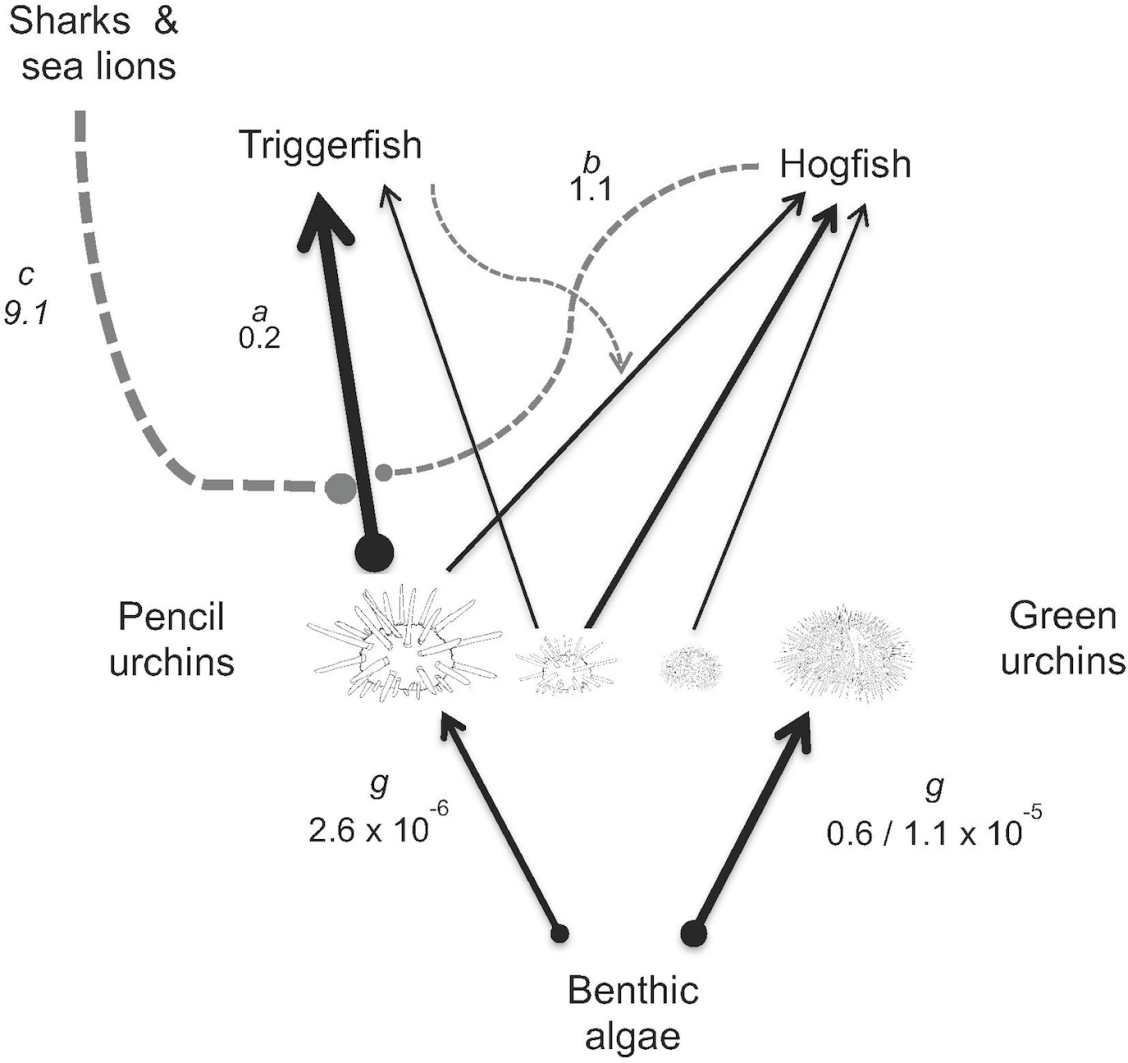
Summary of the density- and behaviorally-mediated interactions characterizing the inferred mechanisms of top-down control in the Galápagos rocky subtidal. Black arrows represent negative interactions with the line thickness roughly proportional to the strength of the interaction. Gray arrows represent a result of the model fitted to pencil urchin survivorship in the TC experiment to quantify hogfish and top-predator interference rates. Dashed lines represent inferred indirect effects. The model parameter *a* represents the per capita attack rate of triggerfish on urchins. Parameters *b* and *c* respectively represent the interference rates with which hogfish and top-predators (sharks and sea lions) affected the strength of the triggerfish-urchin interaction. Parameter *g* represents the per capita grazing rates of the two urchin species. See S4 Table for more information.

This study experimentally demonstrates the key role of consumer species identity in driving a trophic cascade in a speciose food web where the influence of top predators extends to the cascading interactions of algae, urchins and urchin-consumers. In the Galápagos, species identity mattered on two trophic levels of consumers: among mesopredators (fish) and among herbivores (urchins). With 16 species of sea urchin predators known in the Galápagos subtidal (S1 Table), only two species, blunthead and finescale triggerfish, preyed on large pencil urchins to the extent that they precipitated a trophic cascade (Fig 6). Consequently, blunthead and finescale triggerfish are key consumers in the Galápagos subtidal ecosystem, ecologically important for indirect, positive effects on the abundance of benthic algae. In two ways, the identity of sea urchin species mattered similarly and unexpectedly to the strength of trophic cascades, with predatory fish exhibiting strong selection for pencil over green urchins of the same body size, and with pencil urchins exhibiting slower algal grazing rates than green urchins.

Species identity effects may be expected in food webs where strongly interacting species are present, regardless of overall species richness. However, our finding of strong species identity effects on 2 trophic levels in a diverse web is unusual and underscores the need for considering consumer diversity effects across trophic levels [47]. For example, we might have over-generalized the role of predatory fishes in controlling trophic cascades if we had only considered the effects of multiple fish species, instead of multiple urchin species as well, since the trophic cascade depended on one urchin species but not the other. These findings also caution against the aggregation of species with seemingly similar functional roles when using negative associations of predators and herbivores [48], or positive correlations of predators and primary producers [49] to infer top down control or trophic cascades.

In the two decades since Strong’s [9] seminal argument that trophic cascades are *“restricted to low diversity places where great influence can issue from one or few species”* an increasing number of investigations have documented TC’s in species-rich communities, including neotropical insect-plant food webs [50], African savannahs [51] and fish-driven tropical TC’s where the fish—prey link was observed [17, 52] or inferred [53]. Consumer effects in our focal marine benthic system were strong, causing a 24-fold reduction in herbivore densities within 21 hours of the 8-day experiment. Our study therefore suggests that food web diversity per se is not a limiting factor for the existence or strength of trophic cascades.

### Scaling up from experimental results

As all manipulative experiments are interventions in nature [32, 33, 54], it is important to consider how representative the results obtained from field experiments are of the ecosystem at large. Regarding this study, a logical question might be: given that herbivorous pencil urchins are removed by predatory fishes, how valid is the experiment-based inference that a trophic cascade results from a release of herbivore pressure? We assert that this inference is warranted for several reasons. Firstly, nearly all of the trials of four different types of predation experiments conducted in the field (tethering, trophic cascade, size and species selection experiments) and over a span of six years (2007–2013) provided persistent, repeatable results indicating that rapid day-time predation on preferred pencil urchin prey is a predictable characteristic of species interactions in the Galápagos subtidal system. This means that predators can locally release benthic algae from pencil urchin herbivory over the time-scales of daylight hours. We believe these experimental results are robust as they are based on a total of 24.3 days of subtidal experimentation monitored continuously by time-lapse photography or videography to identify urchin predators and key species interactions (234.2 hours of tethering, species and size selection experiments, 350 hours of TC experiments). Secondly, rates of triggerfish predation on pencil urchins were similarly rapid, regardless of the type of substrate that the experiments were performed on, both on exposed algal covered bases (Fig 4) and on the natural rock substrate where potential spatial refuges occurred for the urchins among small rocks and crevices (Fig 2C and 2D, S2E Fig). The intense predation on large pencil urchins by blunthead and finescale triggerfish occurred repeatedly in three different types of experiments performed 5 years apart (2008, 2013) at two sites (Baltra, Baltra South). Thirdly, although spatial refuge was not formally incorporated as a factor in the experimental designs, our experimental measurements of predation on pencil and green urchins were intended to encompass a range of the natural habitat occupancy in the Galápagos subtidal. In nature, these sea urchins occupy a variety of Galápagos subtidal habitats from the top of rock surfaces where they are fully exposed to predators (non-cryptic habitat, Fig 1A) to the interstices of small cobbles or crevices and holes which are partially cryptic habitats [35–38] (J. Witman *unpublished data*). Pencil urchins display all three of these patterns of habitat occupancy while green urchins are mostly non-cryptic on exposed rock surfaces. This breadth of habitat occupancy likely reflects spatial variability in the different drivers of urchin distribution in the Galápagos subtidal, including predation and hydrodynamic forces, which are generally known to affect sea urchin distribution and foraging [15, 55]. The conditions in the open fence treatments of the TC experiments mimicked exposed rock surface habitats with little spatial refuge, while the tethering experiments and species selection experiments on the natural rock substrates allowed partially cryptic behaviors where the urchins could occupy habitats affording some degree of potential spatial refuge from predation. However, potential spatial refuges from triggerfish predation provided by small cobbles and crevices in the substrate during the species selection experiment in particular were ineffective against triggerfish predation as the trigger fish pushed the cobbles aside to eat the pencil urchins (Fig 2D, S2E Fig, J. Witman and F. Smith, *personal observations*). Nonetheless, future experiments in this system should explore the role of spatial variability in urchin habitat occupancy on the strength of the triggerfish-urchin-algae trophic cascade. As suggested by the trend of increased pencil urchin abundance at night, this urchin species appears to exploit a nocturnal refuge from predation, which may explain how they can persist with predatory triggerfish and hogfish, which were only observed during the day. This nocturnal activity mirrors an earlier, unreplicated finding that pencil urchins were more abundant at night than during the day at 3–5 m depth in Academy Bay, Galápagos Islands [38]. Although several other species of sea urchins are nocturnally active [56–58], the extent to which this behavior provides a temporal predation refuge remains largely untested. The site level differences in predation on small pencil urchins was likely due to differences in predatory fish abundance and hydrodynamic forces between the sites. These hypotheses are being investigated by ongoing research.

Overall, our experiments were relatively short, lasting from 4 hours to 8 days. That said, experimental duration in Shurin et al.’s meta-analysis of trophic cascades [59], ranging from 6 to 8030 days, had no significant effect on the response of herbivores or primary producers to predators.

The percent of the substrate on the algal covered bases grazed by pencil urchins in this study was 3.6-fold higher than that recorded on the natural substrate in longer (5 week) experiments with caged pencil urchins at Caamaño Island, another Galápagos subtidal site [37] when both results were normalized to the percent of the substrate grazed per 1.0 m ^2^ per capita per day (0.08% 1.0 m^2^ /urchin/day, [37]; 0.31% 1.0 m^2^ /urchin/day, this study). We attribute the lower grazing rate of pencil urchins at Caamaño to substantially higher wave exposure and flow velocity at Caamaño compared to the Baltra study site where the TC experiments were performed [28].

Although we hypothesized that sea urchins would be involved in trophic cascades, there are other species of herbivores in the Galápagos marine ecosystem, such as marine iguanas, crabs, fish and green turtles [60, 61]. Iguanas and herbivorous crabs forage mostly in the intertidal zone [60]. Although iguanas occasionally feed in the shallow rocky subtidal zone (< 6 m), we never observed them at the depth range of the experiments (10–12 m) at any of the sites over the 6-year study. Green turtles, *Chelonia mydas*, occur at our study sites (Fig 5B), but they never fed on benthic algae, either on the natural substrate or on the algal-covered bases of our experiments. Finally, by virtue of their abundance, yellowtail surgeonfish (*Prionurus laticlavius*) and blue-chin parrotfish (*Scarus ghobban*) likely also influence the distribution of benthic algae in the Galápagos subtidal ecosystem (R. W. Lamb, *personal communication*), thus necessitate future study.

The amount of algal substrate grazed by green urchins was nearly 4-fold higher than that estimated for pencil urchins during the TC experiments. We interpret this as a species-specific difference: green urchins are simply more voracious grazers than pencil urchins. In mesocosm experiments, Carr and Bruno [62] found that the rate of algal consumption by green urchins increased with a doubling of temperature (14.0 vs 28.0°C). Since the average temperatures were colder rather than warmer (by 2.11°C) in the green urchin compared to the pencil urchin TC experiment (S5 Table), temperature is unlikely to explain the difference in grazing rates we observed between the two species. Our experiments suggest that the more voracious green sea urchins are not under top-down control from fish predation, which implies that potential increases in green sea urchin barrens in the Galápagos Marine Reserve are not a consequence of the overfishing of predatory fishes.

### Behavioral indirect interactions

Our study contributes to the understanding of trophic cascades by demonstrating how the strong consumptive effects of a key consumer were altered by the interaction-modifying behaviors of both meso- and top predators in the Galápagos subtidal where both DMII’s and BMII’s are transmitted down to affect the total cover of benthic primary producers (Fig 6). Behavioral interactions among mesopredators were important, with hogfish interference increasing the effective handling time of triggerfish on pencil urchins to the extent that 30 minutes of hogfish interference would prevent the death of 1 urchin from triggerfish predation. Since hogfish only scavenged the remains of large urchins after the triggerfish abandoned the predated urchin and did not successfully attack and consume them directly, we infer that large pencil urchins have an escape in size from hogfish predation. We subsequently observed kleptoparasitic behavior of hogfish at the Baltra site where a hogfish stole a pencil urchin from a finescale triggerfish feeding on it (J. Witman and F. Smith, *personal observations*). As it is focused on small pencil urchins, an additional role of hogfish predation in this system may be to reduce the recruitment of pencil urchins or to restrict small urchins to refuges from predation [38].

Predation risk has been increasingly recognized as an important mechanism of BMII’s, particularly in systems with abundant top-predators [63, 64]. The behavioral effects of sharks and sea lions in our system were estimated to be 8.2-fold higher than the hogfish–triggerfish interaction (Fig 6, S4 Table) such that only 2.44 minutes of top predator presence would eliminate the death of 1 pencil urchin from triggerfish predation. This response to top predators is likely a risk effect, as sharks and sea lions were not feeding on urchins. More *in situ* investigations are needed to quantify the worldwide prevalence of such strong indirect effects linked to sea lions and sharks.

Similar risk effects of fur seals have also been documented in the New Zealand subtidal where they reduced the foraging of herbivorous fish [65]. Although interspecific interference competition is widespread among foraging birds, mammals and insects [66–68] and has been studied for decades, one of the first studies demonstrating how interference can be a mechanism of BMII’s wasn’t published until 2006 [69]. Since there are many agonistic interactions among foraging fish in the tropics [52, 70], further research in this area is needed to address whether interspecific interaction modifications among consumers are an emergent property of speciose food webs, playing just as important role in dampening the strength of trophic cascades as intraspecific interference is hypothesized to play in simple food chains [71].

Despite the substantial body of work on trophic cascades, much work still remains to evaluate variation in cascade strength across food webs of diversity rivaling the real world complexity of tropical and subtropical webs [32, 59]. Few studies have investigated BMII’s in tropical trophic cascade experiments generally [27, 72], but our work suggests that considering both DMII’s and BMII’s is necessary despite its challenges in diverse food webs in particular.

## Supporting information

S1 Fig Photo sequence of hogfish interference with blunthead triggerfish foraging on a pencil urchin during the *Eucidaris* TC experiment on June 25, 2012. Photos are taken at 1 second intervals. Triggerfish has an urchin in mouth in A, but then drops it in C after a close pass from the circling hogfish. Circular bases are 0.31 m^2^ area for scale.
(TIFF)

S2 Fig **Photos of predation on pencil urchins either during the tethering (A-E) or trophic cascade experiments.** A & B illustrate adult hogfish attacking small pencil urchins during tethering experiments at Baltra South (A) and Rocas Gordon site (B). A finescale triggerfish is preying on a large *Eucidaris* urchin in C. during a trial at Baltra South on June 27, 2008. Note the hollowed out urchin remains, a signature of triggerfish predation, and the hogfish nearby. The full time series shows the complete feeding sequence. D. Blunthead triggerfish initiating an attack on a large *Eucidaris* during a trial at Baltra South on July 4, 2008. Results from these 2 trials are graphed in Fig 2C. E. Blunthead triggerfish foraging head down in rock rubble for tethered *Eucidaris* during the first prey selection experiment where *Lytechinus* and *Eucidaris* were tethered side by side and place on the natural rock substrate on July 3, 2012. The heterogeneous rubble did not provide a spatial refuge from triggerfish, which ate over 75% of the tethered *Eucidaris* urchins (Fig 3D). E. A blunthead triggerfish consuming one of the 24 urchins in the *Eucidaris* TC experiment, with a hogfish nearby.
(TIFF)

S3 Fig Photos of top predator effects. A-C. represents a time series taken at 1 second intervals during the *Eucidaris* TC experiment showing interference effects of a diving sea lion. Note that the school of zooplanktivorous scissortail damselfish (*Chromis atrilobata*) are high above the sea floor in A, but start to descend in B, as the sea lion above them dives toward the bottom. C. shows the diving sea lion at the upper left, the descending damselfish school below it, and the blunthead trigger dropping the pencil urchin that it had removed from a treatment (shown in A,B) as it is startled by the diving sea lion. A hogfish moves in to bite the prey remains of the pencil urchin the triggerfish was feeding on. Yellow arrow in A shows location of triggerfish. D. shows a Galápagos shark *Carcharhinus galapagensis* swimming directly over the *Eucidaris* TC experiment. Circular bases are 0.31 m^2^ area for scale.
(TIFF)

S1 Table List of 16 species of known sea urchin predators in the Galápagos Islands. The source of diet information is listed, * representing a direct feeding observation made by J. Witman or F. Smith., 1 = *www.fishbase.org*, 2 = Grove JS, Lavenberg RJ. 1997, 3 = Humann P, DeLoach N. 2002 4 = Dee LE, Witman JD, Brandt. 2012, 5 = *http://www.iucnredlist.org/*, 6 = Martinez C. 2000.
(PDF)

S2 Table Locations of research sites in the Galápagos Islands. (PDF)

S3 Table Observations of behavioral interactions between adult hogfish *Bodianus diplotaenia* and triggerfish preying on tethered pencil urchins *Eucidaris galapagensis* during overnight trials (2008). Data from time lapse images taken at 2 minute intervals. Urchin survivorship data from these trials are plotted in Fig 2C.
(PDF)

S4 Table Summary of parameters of Beddington-DeAngelis functional response model fitted to pencil urchin survivorship in the trophic cascade experiment (Fig 5A) to quantify hogfish and top predator interference rates. The parameters *a* and *h* respectively represent the per capita attack rate and handling time with which triggerfish consume urchins, and the parameters *b* and *c* respectively represent the behavioral interference rates with which hogfish and top-predators affected the strength of the triggerfish-urchin interaction. SE = standard error.
(PDF)

S5 Table Temperature regime during the trophic cascade experiments. Temperatures were recorded at 5 minute intervals throughout each TC experiment by an Onset Tidbit data logger (Onset Computer Corporation, Pocasset, Massachusetts, USA, +/- 0.01°C precision) attached to one of the control bases. The temperature record for the pencil urchin TC experiment ran from 11:50 on June 23, 2012 to 11:40 on July 1, 2012, while the record for the TC experiment with green urchins extended from 12:25 on July 13, 2012 to 12:15 on July 20, 2012.
(PDF)

S1 Video Video illustrating hogfish interference with triggerfish predation on a pencil urchin (*E*. *galapagensis*) during the trophic cascade experiment. It was made from 80 seconds of a triggerfish attack on a pencil urchin in an open (fence) treatment, where an adult hogfish enters the area 25 seconds after the triggerfish. The hogfish swims closely around the feeding trigger at least twice. The video shows the hogfish head down at the sea urchin alongside the feeding trigger. The hogfish then emerges with part of an urchin in its mouth. The triggerfish stops feeding abruptly on the urchin at 72 seconds into the original photo series and begins to swim out of the area while the hogfish continues feeding on the urchin remains.
(MOV)
