# Supplementary figures and images for: Experimental demonstration of a trophic cascade in the Galápagos rocky subtidal: Effects of consumer identity and behavior
Source: PLoS One. 2017 Apr 21;12(4):e0175705. doi: 10.1371/journal.pone.0175705 (PMC5400256; doi:10.1371/journal.pone.0175705)

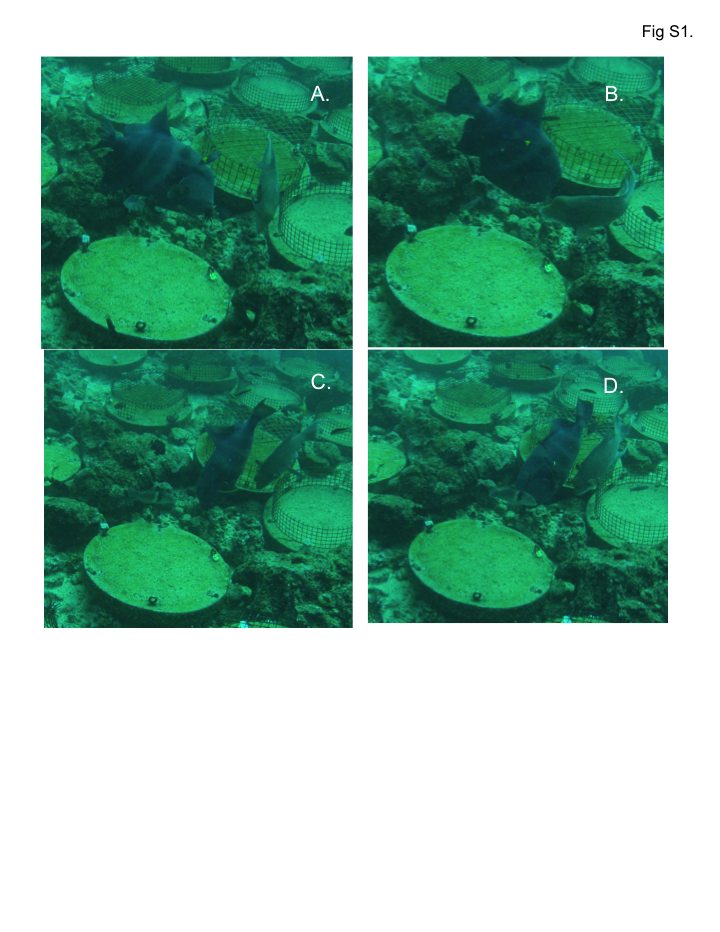

Supplement: S1 Fig — Photos are taken at 1 second intervals. Triggerfish has an urchin in mouth in A, but then drops it in C after a close pass from the circling hogfish. Circular bases are 0.31 m2 area for scale. (TIFF) [file pone.0175705.s001.tiff]

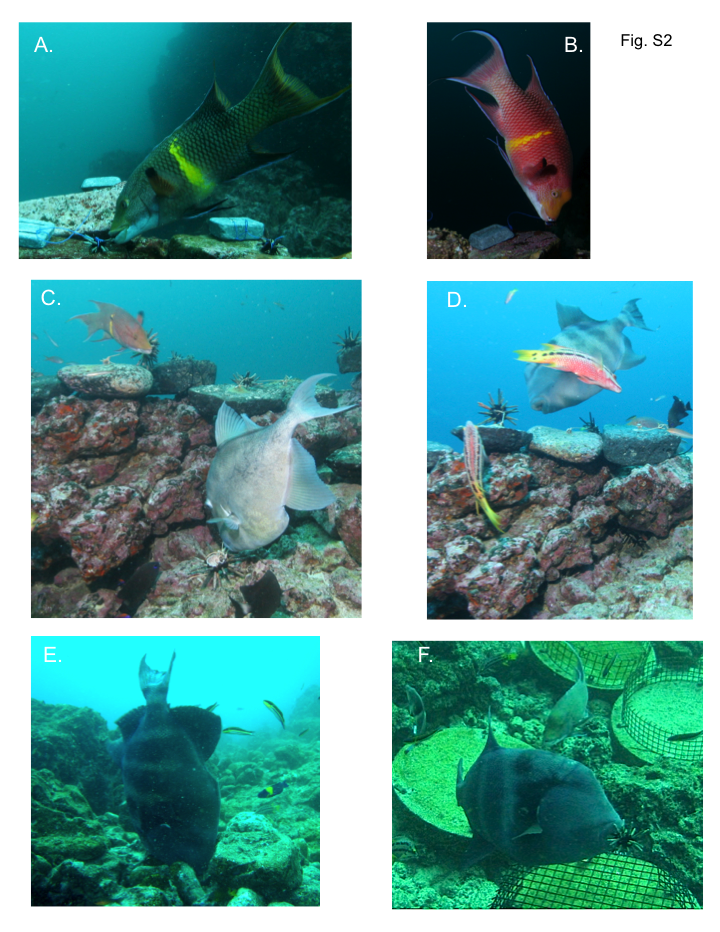

Supplement: S2 Fig — Photos of predation on pencil urchins either during the tethering (A-E) or trophic cascade experiments. A & B illustrate adult hogfish attacking small pencil urchins during tethering experiments at Baltra South (A) and Rocas Gordon site (B). A finescale triggerfish is preying on a large Eucidaris urchin in C. during a trial at Baltra South on June 27, 2008. Note the hollowed out urchin remains, a signature of triggerfish predation, and the hogfish nearby. The full time series shows the complete feeding sequence. D. Blunthead triggerfish initiating an attack on a large Eucidaris during a trial at Baltra South on July 4, 2008. Results from these 2 trials are graphed in Fig 2C. E. Blunthead triggerfish foraging head down in rock rubble for tethered Eucidaris during the first prey selection experiment where Lytechinus and Eucidaris were tethered side by side and place on the natural rock substrate on July 3, 2012. The heterogeneous rubble did not provide a spatial refuge from triggerfish, which ate over 75% of the tethered Eucidaris urchins (Fig 3D). E. A blunthead triggerfish consuming one of the 24 urchins in the Eucidaris TC experiment, with a hogfish nearby. (TIFF) [file pone.0175705.s002.tiff]

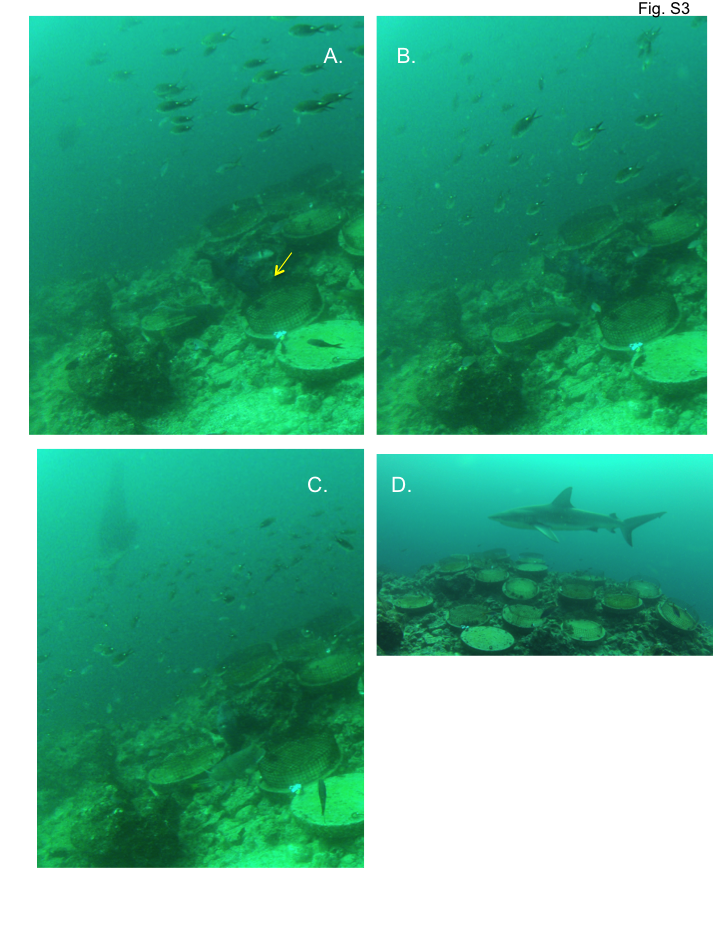

Supplement: S3 Fig — A-C. represents a time series taken at 1 second intervals during the Eucidaris TC experiment showing interference effects of a diving sea lion. Note that the school of zooplanktivorous scissortail damselfish (Chromis atrilobata) are high above the sea floor in A, but start to descend in B, as the sea lion above them dives toward the bottom. C. shows the diving sea lion at the upper left, the descending damselfish school below it, and the blunthead trigger dropping the pencil urchin that it had removed from a treatment (shown in A,B) as it is startled by the diving sea lion. A hogfish moves in to bite the prey remains of the pencil urchin the triggerfish was feeding on. Yellow arrow in A shows location of triggerfish. D. shows a Galápagos shark Carcharhinus galapagensis swimming directly over the Eucidaris TC experiment. Circular bases are 0.31 m2 area for scale. (TIFF) [file pone.0175705.s003.tiff]
