# Supplementary material for: Experimental demonstration of a trophic cascade in the Galápagos rocky subtidal: Effects of consumer identity and behavior
Source: PLoS One. 2017 Apr 21;12(4):e0175705. doi: 10.1371/journal.pone.0175705 (PMC5400256; doi:10.1371/journal.pone.0175705)
Supplement: S1 Table — The source of diet information is listed, * representing a direct feeding observation made by J. Witman or F. Smith., 1 = www.fishbase.org, 2 = Grove JS, Lavenberg RJ. 1997, 3 = Humann P, DeLoach N. 2002 4 = Dee LE, Witman JD, Brandt. 2012, 5 = http://www.iucnredlist.org/, 6 = Martinez C. 2000. (PDF) [file pone.0175705.s004.pdf]

S1 Table. List of 16 species of known sea urchin predators in the Galápagos Islands.

The source of diet information is listed, with * representing a direct feeding observation made by J. Witman or F. Smith., 1,= *www.fishbase.org*, 2 = Grove & Lavenberg (1997), 3 = Humann & DeLoach (2002), P. 4 = Dee et al. (2012), 5 = *http://www.iucnredlist.org/*, 6 = Martinez (2000)

**Fish**		Source
Spanish hogfish	*Bodianus diplotaenia*	, *, *1 - 3*
Harlequin wrasse	*Bodianus elancheri*	*, *2*
Goldspot sheephead	*Semicossphyus darwinii*	*2*
Finescale triggerfish	*Balistes polylepis*	*, *1,3*
Blunthead triggerfish	*Pseudobalistes naufragium*	*, *1,3*
Guineafowl puffer	*Arothon meleagris*	*,
Spot-fin Porqupinefish	*Diodon hystrix*	*, *1*, 2
Balloonfish	*Diodon holocanthus*	*1, 2,*
Pacific burrfish	*Chilomyterus affinis*	*
Spotfin Burrfish	*Chilomyterus reticulatus*	*
Beakfish	*Oplegnathis insignis*	*, *2,*
Yellow bellied triggerfish	*Sufflamen verres*	*3, 5*
**Invertebrates**		
Red spiny lobster	*Panilurus pencillatus*	*6*
Green spiny lobster	*Panilurus gracilis*	*6*
Slipper lobster	*Scyllarides astori*	*6*

Sea star	*Pentaceraster cumingii*	**, 4*

REFERENCES for S1 Table

Dee LE, Witman JD, Brandt M Refugia and top-down control of the pencil urchin *Eucidaris galapagensis* in the Galapagos Marine Reserve. 2012. Journal of Experimental Marine Biology and Ecology.416-417: 135-143.

Grove JS, Lavenberg RJ The fishes of the Galápagos Islands. 1997. Stanford University Press, Stanford, 863 p.

Humann P, DeLoach N Reef fish identification: Galapagos. 2003. New World Publications, Inc. Jacksonville, FL.

Martinez C. Ecología trófica de *Panulirus gracilis*, *P. penicillatus* y *Scyllarides astori* (Decapoda, Palinura) en sitios de pesca de langosta de las Islas Galápagos. 2000 Tesis de Licenciatura, Universidad del Azuay, Cuenca (Ecuador)
