# Supplementary material for: Experimental demonstration of a trophic cascade in the Galápagos rocky subtidal: Effects of consumer identity and behavior
Source: PLoS One. 2017 Apr 21;12(4):e0175705. doi: 10.1371/journal.pone.0175705 (PMC5400256; doi:10.1371/journal.pone.0175705)
Supplement: S2 Table — (PDF) [file pone.0175705.s005.pdf]

S2 Table. Locations of research sites in the Galápagos Islands

Site	Latitude and Longitude	
Isla Champion	S 01.23683 º	W 090.38498 º
Guy Fawkes	S 00.498966º	W 090.51222 º
Rocas Gordon	S 00.56652 º	W 090.14147 º
Baltra	S 00.41100 º	W 090.27525 º
