# Supplementary material for: Experimental demonstration of a trophic cascade in the Galápagos rocky subtidal: Effects of consumer identity and behavior
Source: PLoS One. 2017 Apr 21;12(4):e0175705. doi: 10.1371/journal.pone.0175705 (PMC5400256; doi:10.1371/journal.pone.0175705)
Supplement: S3 Table — Data from time lapse images taken at 2 minute intervals. Urchin survivorship data from these trials are plotted in Fig 2C. (PDF) [file pone.0175705.s006.pdf]

S3 Table. Observations of behavioral interactions between adult hogfish *Bodianus diplotaenia* and triggerfish preying on tethered pencil urchins *Eucidaris galapagensis* during overnight trials (2008). Data from time lapse images taken at 2 minute intervals. Urchin survivorship data from these trials are plotted in Fig. 2C.

Trial Name and Date	Time	Observation
Baltra South 1 June 28, 2008	09:05:06	Blunthead trigger attacking large urchin
	09:07:06	“ “ “
	09:09:06	Hogfish close to trigger feeding on urchin
	09:21:06	Blunthead trigger initating another attack on urchin , continues until 0:9:25
	0:9:23: 06	2 hogfish close to feeding trigger
	0:9:25:06	3 hogfish close to predated urchin, triggerfish in background
	0:9:29:06	1 hogfish feeding on triggerfish predated urchin remains
	0:9:49:06	Another hogfish bites same urchin remains
Baltra South July 3, 2008	11:39:41	Finescale trigger begins predation on urchin
	11:43:41	Trigger still feeding, drags small rock urchin tethered to off ledge
	16: 29:41	Trigger attacks last large tethered urchin

16:31:41	Trigger still feeding on urchin, , hogfish close by
17:47:41	Hogfish scavenging triggerfish predated urchin remains
