# Supplementary material for: Experimental demonstration of a trophic cascade in the Galápagos rocky subtidal: Effects of consumer identity and behavior
Source: PLoS One. 2017 Apr 21;12(4):e0175705. doi: 10.1371/journal.pone.0175705 (PMC5400256; doi:10.1371/journal.pone.0175705)
Supplement: S4 Table — The parameters a and h respectively represent the per capita attack rate and handling time with which triggerfish consume urchins, and the parameters b and c respectively represent the behavioral interference rates with which hogfish and top-predators affected the strength of the triggerfish-urchin interaction. SE = standard error. (PDF) [file pone.0175705.s007.pdf]

S4 Table. Summary of parameters of Beddington-DeAngelis functional response model fitted to pencil urchin survivorship in the trophic cascade experiment (Fig 5A) to quantify hogfish and top predator interference rates. The parameters *a* and *h* respectively represent the per capita attack rate and handling time with which triggerfish consume urchins, and the parameters *b* and *c* respectively represent the behavioral interference rates with which hogfish and top-predators affected the strength of the triggerfish-urchin interaction. SE = standard error.

Parameter	Estimate	SE
a	0.240	0.405
h	2.527	1.084
b	1.086	2.404
c	9.113	24.288
