# Supplementary material for: Experimental demonstration of a trophic cascade in the Galápagos rocky subtidal: Effects of consumer identity and behavior
Source: PLoS One. 2017 Apr 21;12(4):e0175705. doi: 10.1371/journal.pone.0175705 (PMC5400256; doi:10.1371/journal.pone.0175705)
Supplement: S5 Table — Temperatures were recorded at 5 minute intervals throughout each TC experiment by an Onset Tidbit data logger (Onset Computer Corporation, Pocasset, Massachusetts, USA, +/- 0.01°C precision) attached to one of the control bases. The temperature record for the pencil urchin TC experiment ran from 11:50 on June 23, 2012 to 11:40 on July 1, 2012, while the record for the TC experiment with green urchins extended from 12:25 on July 13, 2012 to 12:15 on July 20, 2012. (PDF) [file pone.0175705.s008.pdf]

S5 Table. Temperature regime during the trophic cascade experiments. Temperatures were recorded at 5 minute intervals throughout each TC experiment by an Onset Tidbit data logger (Onset Computer Corporation, Pocasset, Massachusetts, USA, ± 0.01 °C precision) attached to one of the control bases. The temperature record for the pencil urchin TC experiment ran from 11:50 on June 23, 2012 to 11:40 on July 1, 2012, while the record for the TC experiment with green urchins extended from 12:25 on July 13, 2012 to 12:15 on July 20, 2012.

Type of TC Experiment	Average Temperature (°C)	Standard Deviation	n
Pencil Urchin	24.96	0.68	2,304
Green Urchin	22.85	0.15	2,015
